# Gene density homogeneity is disrupted by repetitive DNA Expansion

**DOI:** 10.1371/journal.pone.0361209

**Published:** 2026-10-26

**Authors:** Zhaobo Hu, Sean Chien, Kenzie Laird, Heath Blackmon

**Affiliations:** 1 Department of Biology, Texas A&M University, College Station, Texas, United States of America; 2 Department of Biology and Biochemistry, University of Houston, Houston, Texas, United States of America; 3 School of Biological Sciences, University of Louisiana at Lafayette, Lafayette, Louisiana, United States of America; 4 Interdisciplinary EEB Program, Texas A&M University, College Station, Texas, United States of America; Ben-Gurion University, ISRAEL

## Abstract

The democratization of sequencing has led to a wealth of high-quality genome assemblies from across the Tree of Life. One pattern that has emerged is that chromosomes within a genome can vary drastically in their gene density (e.g., human chromosomes 13 and 19, with gene densities of 17.5 and 60.5 genes per megabase, respectively). To our knowledge, the cause of this variation in gene density has not been explored. In this study, we leveraged genome assemblies of 262 vertebrate species to examine the range of within-genome variation in gene density. We combined gene density data with repeat landscape analyses in each species. We predicted that genomes with more recently expanded repeat families should show greater variation in gene density, because repetitive elements accumulate unevenly across the genome, while chromosomal rearrangements such as fusions, fissions, insertions, deletions, and inversions homogenize gene density over time. Our empirical analysis reveals a significant yet variable effect of repeat expansions, with additional processes likely contributing to variation in gene density.

## Introduction

The degree of gene density homogeneity across chromosomes varies widely among species, yet the evolutionary processes that shape this genomic feature remain largely unexplored. Genome architecture can differ markedly across species, and even within a single genome, chromosomes often show striking differences in size and gene content. In *Homo sapiens*, for example, chromosomes 13 and 19 (114.4 vs. 58.6 Mb, respectively) differ by a factor of 3.46 in gene density, with 2000 genes on chromosome 13 and 3547 on chromosome 19 [1]. Similar patterns are observed across vertebrates: in *Mus musculus*, gene density varies more than twofold across chromosomes [2], and across Sauropsida, compact microchromosomes are consistently more gene-dense than larger macrochromosomes [3]. These widespread departures from homogeneity raise the question of what evolutionary forces promote or disrupt uniform gene density across chromosomes. Because gene density is closely linked to chromatin organization, recombination, and mutation rates [4–6], understanding its intragenomic distribution is crucial for uncovering the mechanisms that shape genome architecture and function. Although comprehensive comparative analyses across taxa are scarce, available genome assemblies typically show a significant linear increase in gene count with chromosome size. Gene density varies from one chromosome to another, and the magnitude of this variation between chromosomes differs across genomes (Fig 1).

**Fig 1:**
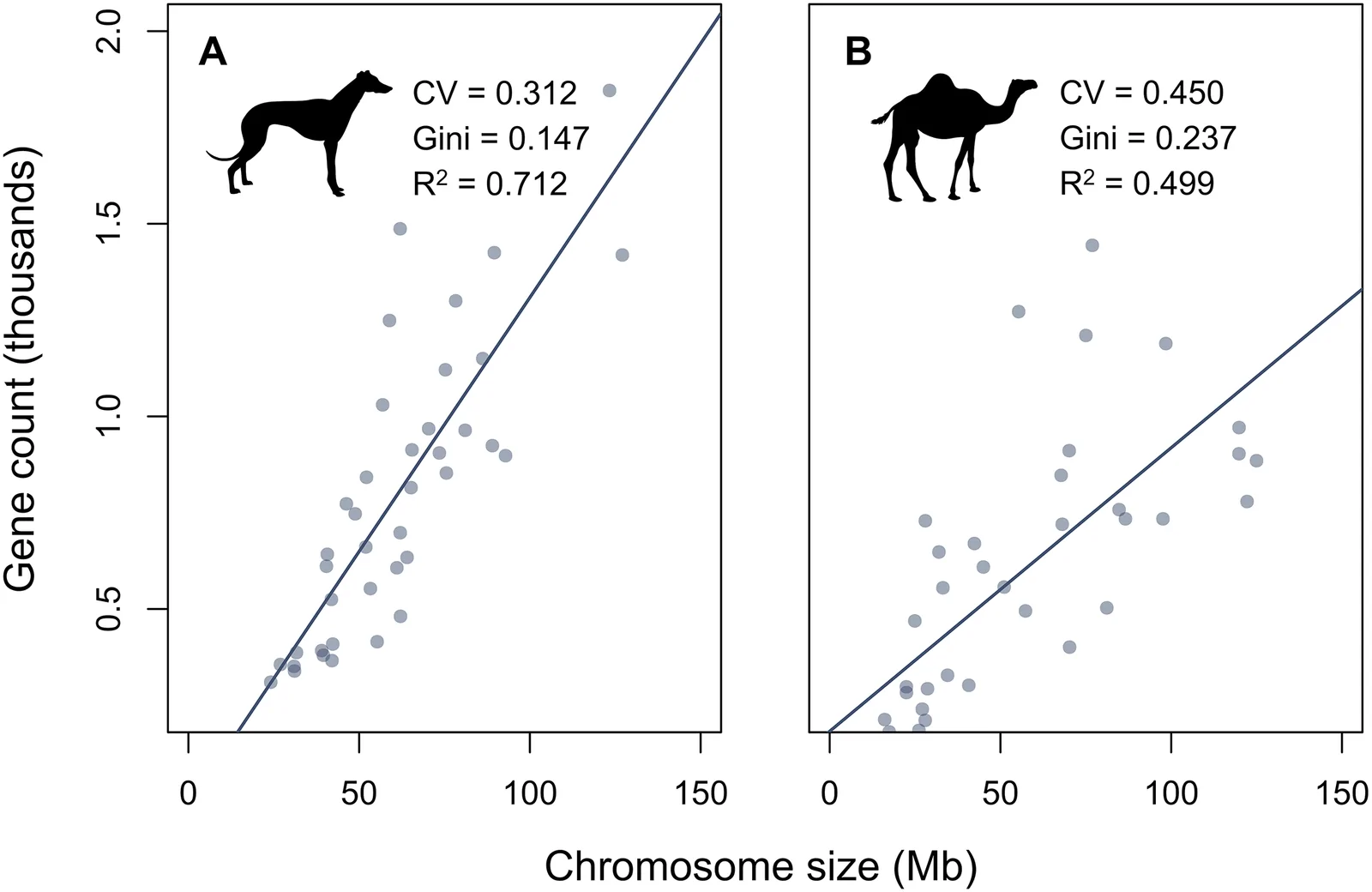
Chromosome size vs gene count for all chromosomes in *Canis lupus familiaris* (A) and *Camelus dromedarius* (B). For each species, trendlines depict least-squares regressions in which chromosome size predicts gene count. (A) Chromosomes in the *Canis lupus familiaris* genome exhibit less deviation from the trendline than those in the (B) *Camelus dromedarius* genome, indicating greater uniformity in gene density within the former genome. The image silhouettes are from PhyloPic [7] and were contributed as follows: *Canis lupus familiaris* by T. Michael Keesey (2012, CC0 1.0 Universal) and *Camelus dromedarius* by Katy Lawler (CC0 1.0 Universal).

To explore how gene density evolves, it is important to consider the processes that can alter its uniformity across chromosomes. Indels are one such process; deletions that remove intergenic or noncoding regions compress genes into a smaller space, thereby increasing density, while insertions expand noncoding sequences and dilute gene packing. Because heterochromatic, gene‑poor regions are repaired less efficiently, indels tend to accumulate there [8]. In contrast, purifying selection quickly removes disruptive indels in gene‑rich areas, preserving their sequence length [9, 10]. A widespread deletion bias then drives a preferential loss of noncoding DNA, resulting in a greater proportion of deletions in chromosomes with larger noncoding regions over time [11, 12]. Together, these forces shrink gene-poor chromosomes faster than gene-rich ones, causing both to approach the genome-wide average and thereby reducing variation among chromosomes.

Chromosomal fissions and fusions break and rejoin whole chromosomes, mixing gene-rich and gene-poor regions. As these rearrangements shuffle coding and noncoding blocks, extreme density differences between chromosomes will gradually decrease. For instance, the highly rearranged genome of *Nomascus leucogenys* shows intermingled GC-rich and GC-poor tracts on the same chromosomes, resulting in more uniform gene densities [13]. Through repeated fissions and fusions, each chromosome’s gene density converges on the genome‑wide mean, reducing overall variance.

In contrast to the homogenizing effects of indels and rearrangements, repeat expansion can increase variation in gene density by adding large amounts of noncoding DNA unevenly across the genome. Transposable elements and satellite repeats often accumulate in hotspots, where they disproportionately inflate the length of some chromosomes or regions relative to their gene content [14]. As a result, uneven repeat expansion can amplify variation in gene density, particularly when purifying selection is weak.

These observations raise an essential question: What balance of forces drives some species toward uniform gene densities while others diverge dramatically? Although the molecular behavior of individual TE families is well characterized within genomes, it remains untested whether these properties predict chromosome-scale patterns of gene density variation across species. We hypothesize that gene density variation arises when repetitive elements proliferate more rapidly in genomic hotspots (e.g., pericentromeric regions, open chromatin zones), generating long expanses of noncoding sequence that amplify gene density variation across chromosomes. In the absence of repeat expansion, mutations and chromosomal rearrangements are expected to gradually homogenize gene density across chromosomes. Although we do not explicitly test for homogenization via rearrangements, our study quantifies the correlation between the extent of repeat expansion and species-level patterns of gene density variation.

## Materials & methods

### Data collection

We downloaded GTF and unmasked FASTA files for all vertebrate species available in the Ensembl Genome Browser version 113 [15]. We also downloaded a chordate species tree from TimeTree 5 for phylogenetic analysis [16, 17].

### Genome selection

We used an R script to obtain a measure of gene density uniformity from sequence and annotation files. For each species, we recorded the name and size of every sequence in the assembly. Where an assembly contains at least three named chromosomes, we retain all named chromosomes. Otherwise, we retain the 60 longest sequences with at least 10Mb; we imposed these limits to exclude low-quality sequence fragments, but we believe that the resulting sequence set is still likely to include most chromosomes in a well-assembled, scaffold-level vertebrate genome, including microchromosomes under ~23Mb [18]. We did not impose a lower limit to sequence size in chromosome-level assemblies, but we excluded mitochondrial sequences. We then excluded species with fewer than three remaining contigs to ensure robust inference of gene density using linear models. Finally, we excluded species whose captured contig sizes totaled less than 80% of the assembly, as these likely contain excessive fragmentation or unassembled repetitive sequences. While these thresholds are largely subjective, they were chosen to balance the challenges of noise in our data with the goal of maximizing the amount of retained data. Using GTF files, we recorded the number of genes corresponding to each captured contig. We fit a linear model in which the size of the retained contigs was the predictor variable and the number of genes on each contig was the response variable. For each species, we present three measures of gene density homogeneity. First, we use the coefficient of variation (CV) for gene density and reverse the sign. CV serves as a direct measure of dispersion independent of linearity. Second, we compute the Gini coefficient for gene density, weight by contig size, and reverse the sign of the resulting value. The Gini coefficient measures the inequality of the distribution among chromosomes, where values near 0 indicate even distribution while values near 1 represent extreme Lorenz curves where one or few chromosomes/contigs contribute all of the gene density variation. Third, we compute the coefficient of determination (R^2^) from linear model fits, in which the size of the retained contigs predicts the number of genes on each contig. R^2^ ranges from 0 to 1, with greater values indicating a better fit to a linear relationship between chromosome size and the number of genes on each chromosome. Although the interpretation for each measure of gene density variation differs, higher values should indicate greater homogeneity on all three measures.

### Repeat evaluation

A repeat masking pipeline [19] was used to identify repeats and calculate repeat divergence for each remaining genome. We built assembly-specific repeat libraries by creating BLAST-compatible databases via RepeatModeler’s BuildDatabase script [20], followed by RepeatModeler v2.0.5 with the -quick setting. The assembly-specific libraries were then combined with family-specific libraries using the RepeatMasker utility script *famdb.py*, which retrieves consensus models for each repeat family from Dfam v3.7 and NCBI Taxonomy [21, 22]. RepeatMasker v4.1.5 was used on the combined library with the flags *-qq* and *-a* [23]. The alignment files were used in the RepeatMasker utility script *calcDivergenceFromAlign* to generate a summary file for each species containing repeat content per repeat class per divergence score for each repeat class and divergence score.

We condensed repeats found by RepeatMasker into six broad categories: LTR retrotransposons, LINE retrotransposons, SINE retrotransposons, DNA transposons, unidentified repeats, and repeats not annotated in every species (“Others”; this category includes simple repeats, satellites, and various RNA repeats). We calculated the proportion of the assembly size represented by each repeat family in a given genome and the median Kimura divergence score of the repeats in each class. We interpret this divergence as a proxy for the timing of repeat expansion within lineages. We normalized each predictor to a range of 0–1 using min-max scaling prior to model fitting. Because the scaled predictors span exactly one unit, the resulting β coefficient can be interpreted directly as the estimated change in the response variable given a change across the entire range of the observed predicted variable.

We predicted a negative effect of repeat proportion on gene density homogeneity, a positive effect of repeat sequence divergence, and a positive effect of the divergence×proportion interaction. A positive interaction estimate indicates that the two main effects reinforce each other rather than canceling out. As the proportion of repeat nucleotides increases, we expect the positive estimate for sequence divergence to increase because additional repeats increase the opportunity for chromosomal rearrangement to decrease gene density. Similarly, as the age of the expansion increases, we expect the negative estimate of the proportion to shift toward the positive, reflecting the reinforcing effect of high sequence divergence on gene density uniformity. Under these predictions, gene density homogeneity is highest in genomes with few repeats and predominantly old repeats, whereas homogeneity is lowest in genomes with many repeats and predominantly young repeats.

### Model fitting

For each dataset, including all species, Mammalia, Actinopterygii, and Sauropsida, we built linear models where CV, Gini coefficient, and R^2^ are explained by repeat sequence divergence and volume. Six types of repeats were considered as predictor variables: DNA transposons, LINEs, LTR transposons, SINEs, unidentified repeats, and all other repeats identified by the previous pipeline. For each repeat type, we included as predictor variables repeat sequence divergence, repeat volume, or the interaction between divergence and volume of the same repeat type, yielding a total of 18 possible predictors. Within each dataset, we generated all possible models (up to 15,625) using unique subsets of the 18 predictor variables, with the constraints that (a) interaction terms cannot be present without their constituent main effects and (b) the number of predictors must allow for one residual degree of freedom. As an exception, we excluded all interaction terms from the Sauropsida model space to allow sufficient degrees of freedom given our limited sample size. We then sorted the models by their AICc weights and selected the smallest set of top models whose cumulative AICc weight reached 95% as our confidence set [24]. We tested each submodel for phylogenetic signal (Pagel’s λ) in its residuals and found no clade with more than half of the models in the confidence set showing significant results (S3 File); hence, we present simpler non-phylogenetic generalized least-squares fits. We model-averaged within our confidence sets and considered a predictor as significant if its relative importance was at least 0.5 and its averaged confidence interval did not include zero.

We also tested the effect of divergence and proportion of all repeats, regardless of repeat class. Because these aggregate predictors are linear combinations of the predictors used in the main analysis, including them in the main analysis introduces multicollinearity. Thus, we perform this test in a separate but similar analysis, in which the candidate model set includes all valid combinations of divergence, proportion, and their interaction terms. As in the main analysis, models were ranked by AICc, the 95% confidence set was retained, and model-average coefficients and confidence intervals were computed separately for each clade and each response variable.

To identify and account for highly influential species in our main analysis, we computed Cook’s D for each observation using the top-ranked model. Cook’s D describes the total change in fitted values when a single observation is removed, providing a measure of each species’ impact on model estimates. For each clade and response variable, we removed the top 10% of species by Cook’s D. We then repeated our main analysis on the reduced dataset, refitting all valid candidate models and computing model-averaged coefficients from the 95% dataset.

We assessed model adequacy for each dataset by comparing the observed β coefficients with a distribution of β coefficients generated from permuted data. For each dataset, we performed 2,000 iterations, in which we permuted each predictor, fit our model as with our main analysis, and recorded the β coefficients. We then assessed whether the observed estimates lie within the 95% confidence interval of the permuted samples.

Finally, we directly test the effect of the proportion of young TEs on within-genome homogeneity of gene density. For each species, we defined young TEs as all TEs below a divergence cutoff, and we repeated our analysis across ten cutoffs from 2% to 20% in 2% intervals. At each cutoff, we tested the association between TE proportion and each of the three response variables within each dataset. For each combination of divergence cutoff, dataset, and response variable, we consider two models: one in which gene density homogeneity is explained by a null intercept, and one in which it is explained by the proportion of young repeats in the genome. We rank these models by AIC and assess their significance as with our main analysis. As before, we predict that higher proportions of young repeats would be associated with lower gene-density homogeneity, and that this relationship should weaken as the divergence cutoff is increased to include older repeats, thereby diluting the signal from the young repeats.

## Results

We assembled a dataset of 262 vertebrate genomes spanning Mammalia, Actinopterygii, Sauropsida, and other clades (S1 File). Measures of gene density homogeneity were computed for 117 species. Chromosome assignment was applied for 101 of the 117 species, while the remaining were selected by size. Two species (Tetraodon nigroviridis and Microtus ochrogaster) carried named chromosomes but were excluded because those sequences covered less than 80% of the assembly. Repeat landscape data were available for 187 species, while 104 species had both repeat metrics and measures of gene density homogeneity. Among the 73 assemblies for which BUSCO scores were available on NCBI, seven scored below 95% completeness, and none fell below 90%, indicating an acceptable level of completeness for gene discovery.

Gene density homogeneity varied widely both within and across our three main clades (Fig 2). Sauropsid genomes show the highest median Gini coefficient (0.22) and highest median CV (0.60), indicating the least even distribution of gene density among chromosomes, yet they also show the highest median R^2^ of any clade (0.94), reflecting a highly linear relationship between chromosome size and gene count. The presence of gene-dense microchromosomes likely contributes to this pattern, since these chromosomes are outliers in gene density but contribute little to linearity because of their small size. Actinopterygii show the opposite pattern, with the lowest CV (median 0.13) and Gini (0.07) of any clade, but having an intermediate R^2^ value (0.74). Mammalia exhibits values intermediate among the three clades. Although standard ANOVA shows strong support for between-clade differences in mean gene density homogeneity, phylogenetic ANOVA [23] finds no evidence for among-group differences after accounting for their evolutionary relationships (S2 File).

**Fig 2:**
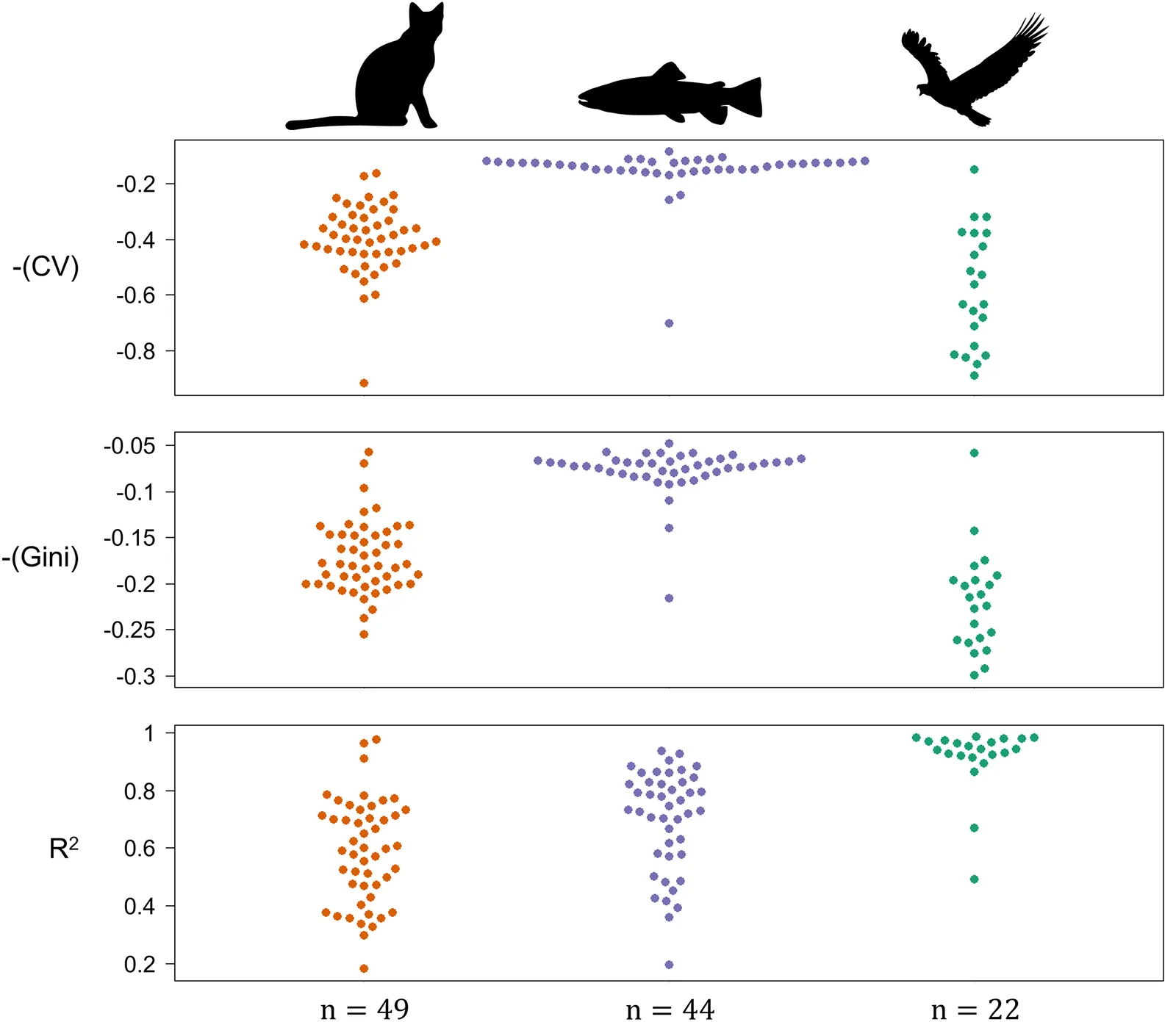
Comparing distributions of gene density homogeneity across three major clades. Beeswarm plots of -(CV), -(Gini), and R^2^ colored by clade. The image silhouettes are from PhyloPic [7] and were contributed as follows: *Felis silvestris catus* by David Orr (2012; Public Domain Mark 1.0); *Salmo trutta* by Carlos Cano-Barbacil (2022; CC0 1.0); and *Haliaeetus leucocephalus* by Steve Hillebrand/U. S. Fish and Wildlife Service (source photo), T. Michael Keesey (vectorization) (2013; Public Domain Mark 1.0).

Across all species, the interaction between repeat sequence divergence and volume in SINE retroelements was the most robust predictor of gene density homogeneity, with -(CV), -(Gini), and R^2^ all reaching consensus on significance and direction (Fig 3; β = 1.49; 0.80; 1.57, respectively). The strongest contributor to this pattern is Mammalia, with a β coefficient of 2.38 for R^2^. However, we find no other significant SINE interaction terms across all three clades and all three response variables. Actinopterygii is represented by 33 species and yields three predictors supported by our hypothesis: the proportion of SINEs against R^2^ (β = 0.41), the interaction between DNA transposon divergence and proportion against Gini (β = 0.36), and the proportion of other repeats (β = −0.34 for R^2^, −0.08 for Gini). Overall, we find mixed support for our hypothesis: across all datasets and response metrics, nine of the 23 supported predictor-dataset combinations had effects on gene density variation that were opposite to our predictions. However, six of these nine were concentrated in the all-species analysis, and we only encountered one such predictor in each clade-specific run. In the clade-specific analyses, we did not observe any cases of disagreement in the direction of the predictor effect across our three response variables.

**Fig 3:**
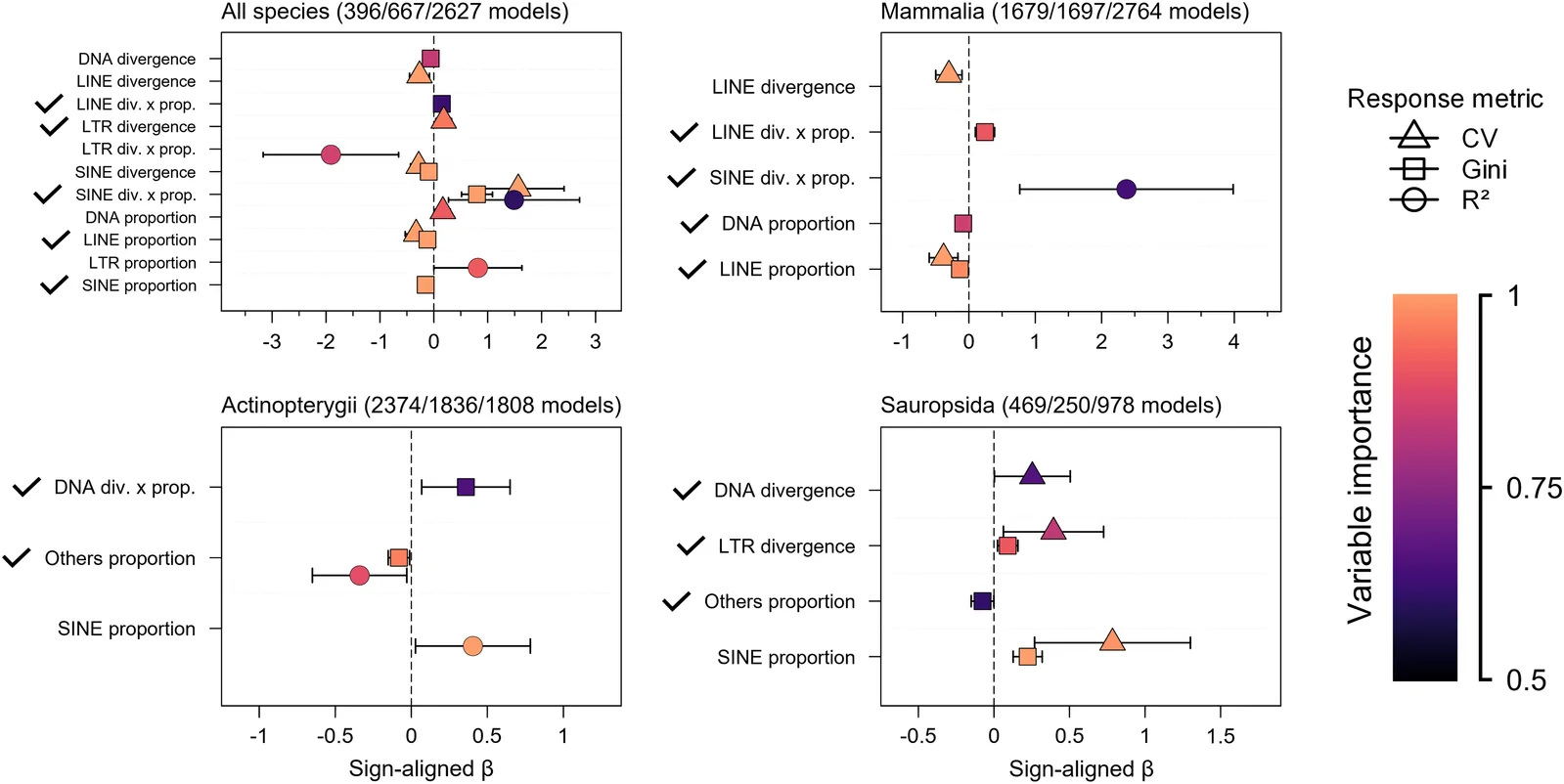
Model-averaged estimates of significant predictors of gene density homogeneity across four datasets. Each panel displays the sign-aligned β estimates for predictors retained in the 95% confidence set of models. Numbers in parentheses describe the number of models retained in each confidence set for -(CV), -(Gini), and R^2^ respectively. Estimates are colored by variable importance. Whiskers represent 95% confidence intervals. The shape of each point indicates the response variable. Check marks to the left of each predictor indicate that the β estimate is consistent with our hypothesis.

Our standalone analysis, using all repeats as predictors, generally yielded very weak predictions for gene density homogeneity (S1 Fig), suggesting that within-genome variation in gene density is primarily driven by specific repeat categories. However, there are two exceptions. First, across all species, with R^2^ as the response variable, the interaction between repeat sequence divergence and proportion has a β coefficient of −1.18, which is an extreme deviation from our prediction that the interaction term should be positive. Second, all three response variables converged on significance with the median sequence divergence of all repeats as the predictor, but no consensus was reached regarding the effect’s direction, with a positive β in R^2^ and negative βs in -(Gini) and -(CV). Overall, these patterns illustrate that aggregating across repeat classes can produce associations that are absent or even reversed in the class-specific analysis.

The removal of the top 10% species by Cook’s D had mixed modeling outcomes (Fig 4). Across all four datasets, removal may change the support for individual effects but never their direction, with all 20 estimates supported in both analyses having signs unchanged. Overall, 16 of the 23 supported combinations were retained after excluding influential species. However, species removal introduces several new significant predictors that were not significant in the full dataset, including LINE divergence, SINE proportion, and the DNA transposon interaction, indicating that some associations are masked by the presence of influential species. In the dataset containing all species, removing 11 influential species retained eight of 11 supported combinations, with the SINE interaction supported under all three measures in both analyses. In Mammalia, the removal of five species cost support for DNA transposon and LINE proportion under Gini, but introduced support for SINE proportion under R^2^. In Actinopterygii, removing four species introduced the interaction between divergence and proportion in the “Other” repeats category and the proportion of other repeats under CV. In Sauropsida, the removal of three species retained only two supported combinations and introduced three more, suggesting that the signal is fragile and driven by a small number of species. The identities of all excluded species are reported in S4 File.

**Fig 4:**
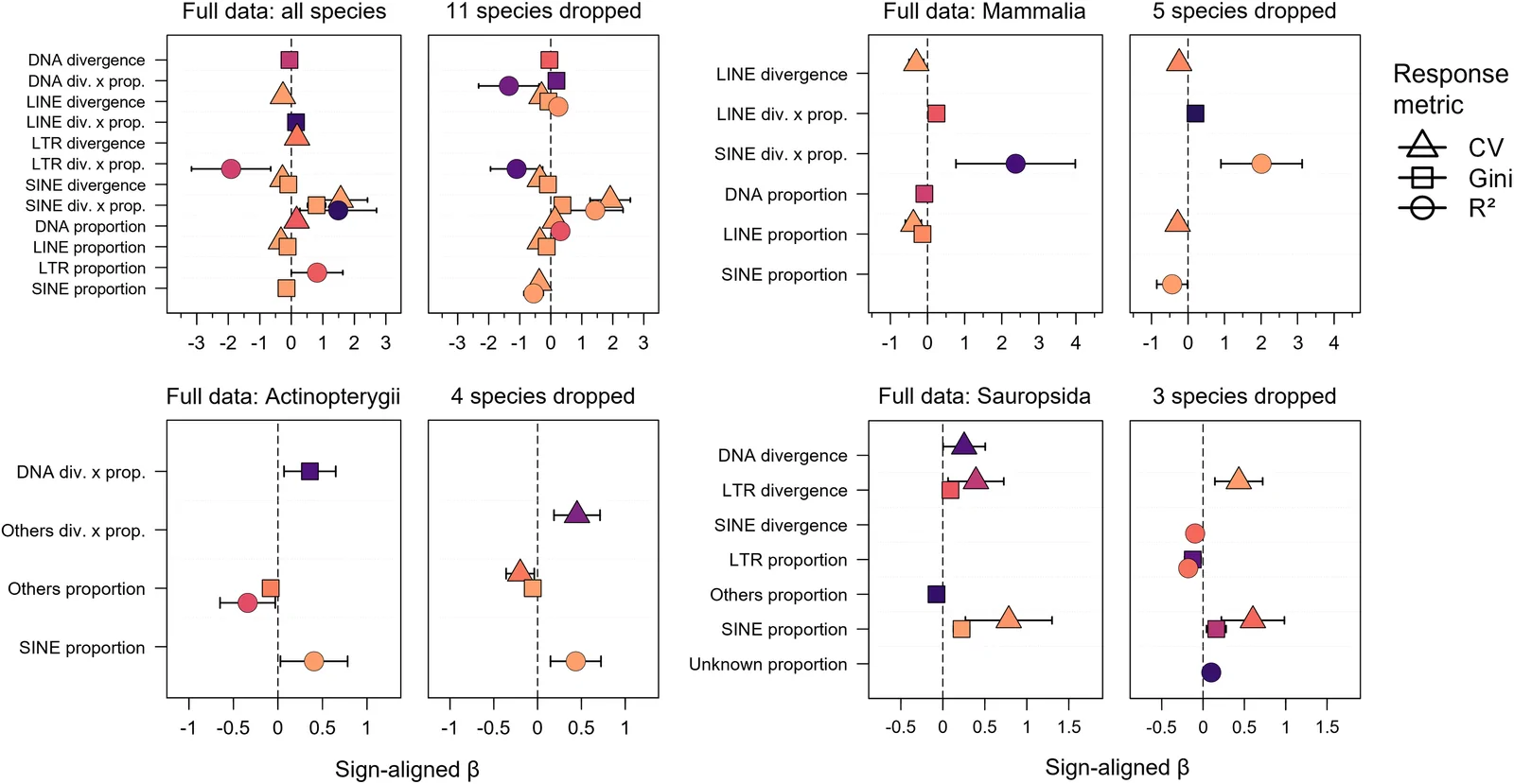
Sensitivity of model-averaged β coefficients to the exclusion of influential species. For each dataset, the left panel reproduces the full-data results from Fig 3, while the right panel shows the β estimates after excluding the top 10% species by Cook’s D. The number of species removed is indicated in the panel title. Only significant predictors are shown.

Of the 31 supported β estimates, 21 fall outside the 95% null interval generated by 2000 predictor permutations (Fig 5). All six supported estimates in Mammalia, as well as eleven of the fifteen in the all-species analysis, lie outside their null intervals. In Actinopterygii, only two of the four supported predictors have effect sizes that exceed the null. In Sauropsida, only the proportion of SINEs against CV and Gini exceed the null; we therefore limit our interpretation of the sauropsid results. Although the permutation intervals are not always centered at zero, we emphasize that under our methods, both the observed and null β coefficients are conditional on inclusion in the 95% cumulative-weight confidence set, and that the observed asymmetry is likely an artifact of the models retained in the confidence set.

**Fig 5:**
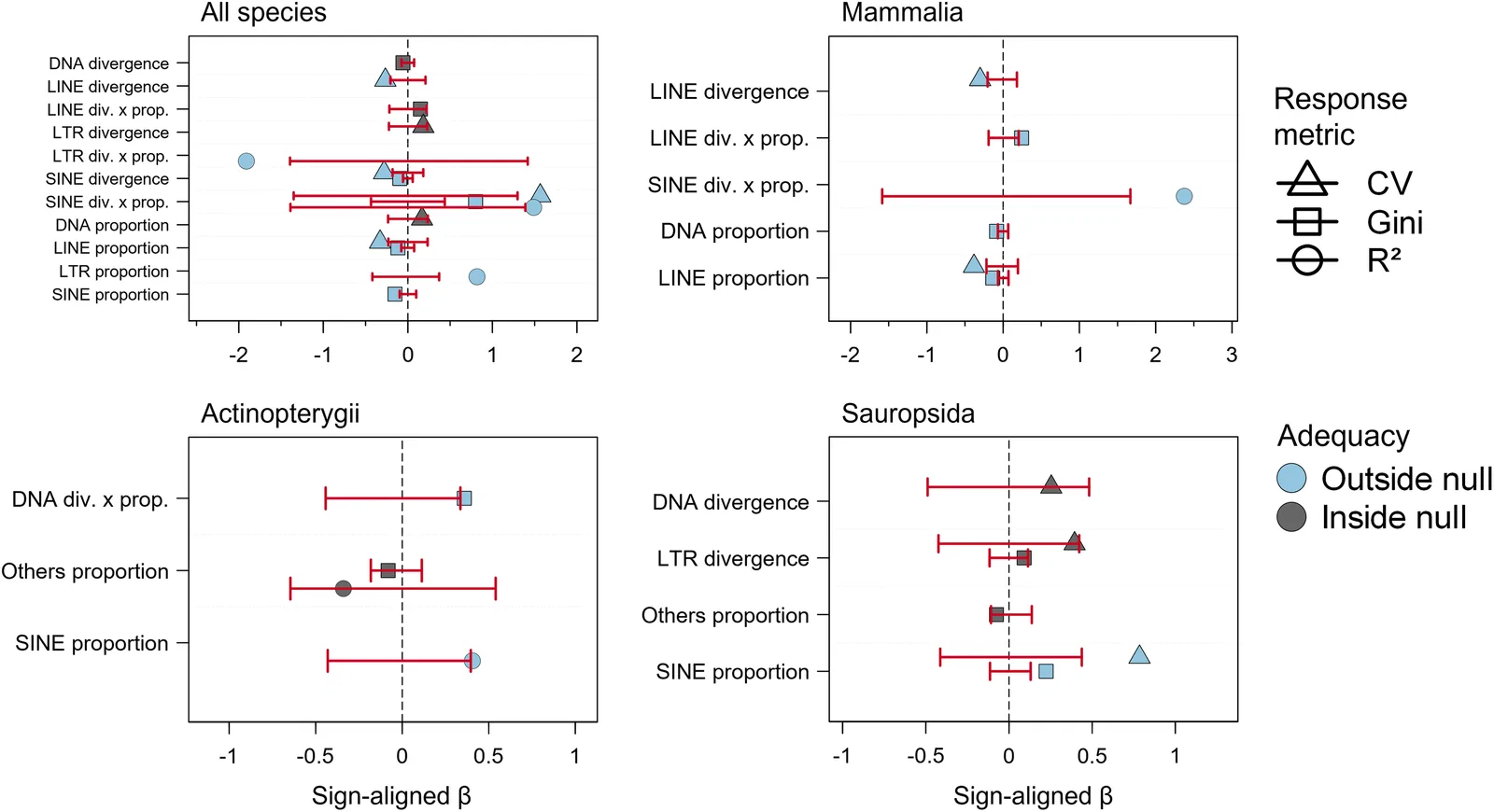
Assessment of model adequacy via predictor permutation. Each panel displays the observed model-averaged β coefficients (points) alongside the 95% confidence interval of the null distribution (red bars) obtained by permuting each predictor variable 2000 times and refitting the models. Point shape represents the response variable; point color indicates whether the point lies beyond its null interval.

In our direct test of the effect of young repeat proportion on gene density homogeneity, we find in Mammalia and Sauropsida that the significant β estimates are all negative, consistent with our prediction (Fig 6). However, as we increase the divergence cutoff in Mammalia and Sauropsida, we observe a weak decline in effect size for R^2^. This suggests that the youngest repeats exert an outsized influence on variation in gene density, whereas the inclusion of older repeats has little additional influence on β estimates. Across all species, by contrast, the proportion of young repeats is positively associated with gene density homogeneity under both Gini and CV at every cutoff examined. This pattern may be driven by Actinopterygii, where β estimates are positive at all cutoffs under all three measures, though supported only for CV at the 16% cutoff.

**Fig 6:**
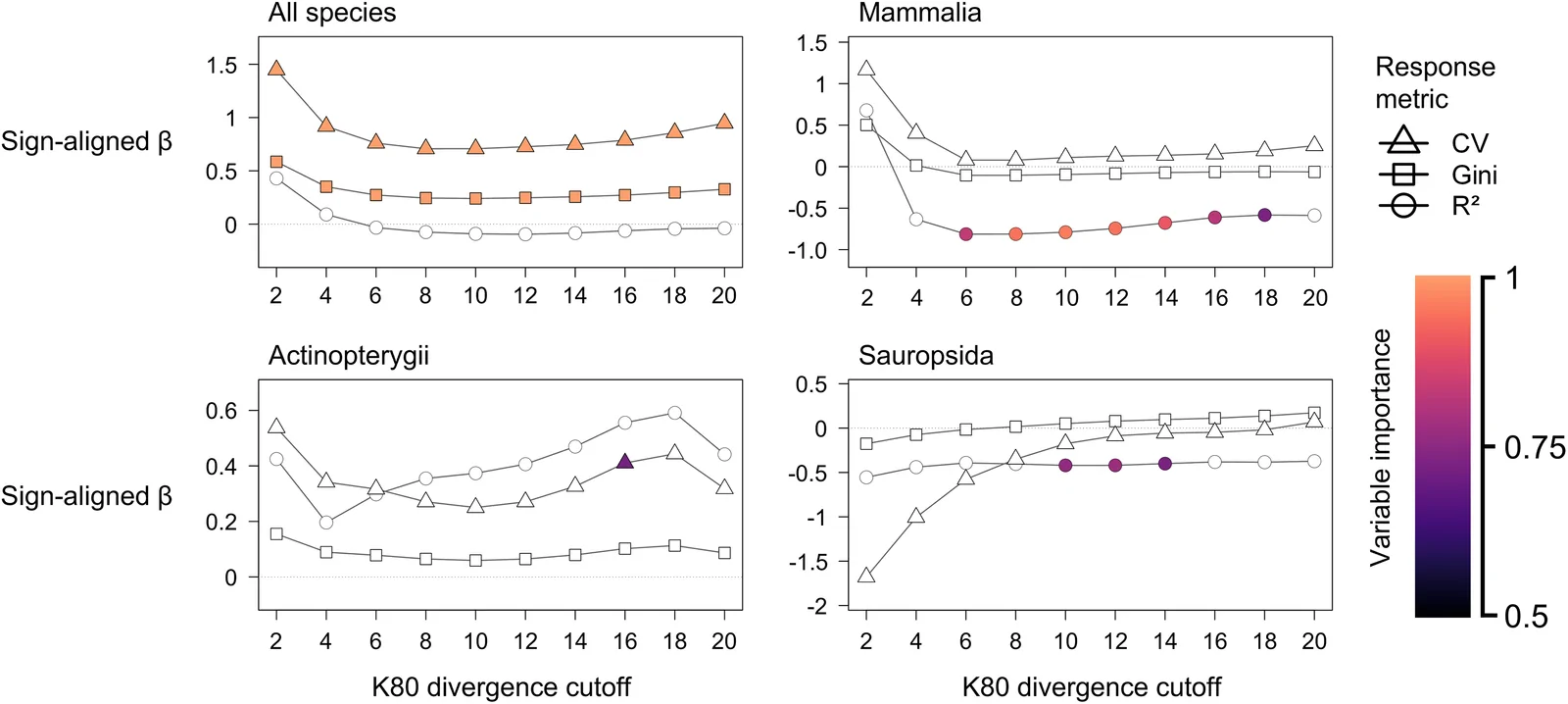
Effect of divergence threshold on the association of aggregate repeat parameters and gene density homogeneity. At each divergence threshold, we tested models with aggregate repeat parameters: the proportion of all repeats within the threshold, their median divergence, and the interaction between these terms.

## Discussion

Our comparative analysis reveals that repeat dynamics play a nuanced role in shaping chromosome-scale gene density. The interaction between SINE divergence and proportion emerged as the most robust predictor of gene density homogeneity across all species, but this signal is driven primarily by Mammalia. Across all datasets and response metrics, the inferred effects on gene density variation vary greatly in size and direction. Nonetheless, the direction of each predictor was consistent across all three response variables.

Nine of the 23 supported predictor-dataset combinations had β coefficients that were opposite to our original predictions, indicating that, for some repeat classes, gene-density homogeneity may be associated with younger and/or less abundant elements. It is important to note that these “effects” reflect statistical associations and do not necessarily imply causal relationships between repeat parameters and gene-density homogeneity. Thus, it is possible that gene density homogeneity is driven by unmeasured genomic features that covary with repeat sequence divergence or proportion.

The direction and magnitude of our SINE divergence × proportion predictor are consistent with the genomic distribution of SINE retroelements in Mammalia. The positive β coefficient indicates that the disruptive effect of SINE abundance on gene density homogeneity is strongest when SINEs are young and weakens as SINEs diverge. One potential explanation is that old SINEs may be experiencing purging from functionally constrained regions. This is consistent with observations in the bovine genome, where young, ruminant-specific repeat families are positively correlated with gene density as they preferentially insert into gene-rich regions, whereas older, ancestral repeats show the opposite pattern [25]. Our divergence threshold analysis reinforces this interpretation: considering repeats beyond the 2% divergence threshold yields negligible changes in effect size.

The comparatively weak signal in Actinopterygii suggests that repeat expansion alone is insufficient to account for homogeneity in within-genome gene density. Amniote genomes exhibit strong patterns of gene-rich and gene-poor chromosomal compartments [26], and this structure underpins the euchromatic accumulation bias observed for SINEs [27]. In contrast, teleost genomes are compositionally homogenous and lack the structure that underlies TE compartmentalization as seen in amniotes [28, 29]. Without structural compartmentalization, transposable elements may not exhibit differential accumulation, resulting in a lack of signal in cross-species analysis. Additionally, the teleost-specific whole-genome duplication and subsequent rediploidization [30] may have reorganized patterns in genome architecture independently of repeat dynamics, further obscuring any underlying associations.

Non-allelic homologous recombination may offer one explanation for the negative effect of repeat sequence divergence on gene density homogeneity. When recombination occurs between highly identical repeats, repeat sequence divergence may bias the mechanism resolving double Holliday junctions (dHJs). As repeats age and diverge, joint‐molecule interfaces become unstable: branch migration and dissolution are less efficient [31], while structure-selective nucleases and resolvases preferentially cleave dHJs into crossover copy number variants [32]. If non-allelic crossovers occur between chromosomes, they could generate translocations, thereby increasing gene-density uniformity by mixing gene-rich and gene-poor sequences. Conversely, dHJ dissolution and non‐crossover gene conversion in young repeats are favored due to high sequence similarity [33]. This method repairs breaks without altering gene counts, thereby leaving gene density homogeneity largely unchanged. Furthermore, old repeats recruit DNA methylation and heterochromatin marks that spread to adjacent genes, thereby silencing or eroding them and amplifying local gene count disparities [34]. If this process acts nonuniformly across chromosomes, it could amplify local variation in gene density and reduce its homogeneity. High methylation at old repeats may also slow branch migration, extending the timeframe for dHJ cleavage before helicases resolve the junction [35]. While these explanations are consistent with observed cases where repeat divergence negatively affects gene density homogeneity, the proposed connection between dHJ resolution and gene density patterns remains to be tested directly.

The clustering of gene density homogeneity in Sauropsida and Actinopterygii may reflect uneven taxonomic representation across Vertebrata, which limits our ability to generalize these patterns to poorly sampled lineages. Only four non‐avian reptile genomes pass our assembly quality thresholds, so the majority of reptilian data comes from Aves. Early-branching groups, such as Agnatha and Chimaeriformes, were excluded due to low contig recovery, despite their potential to illuminate ancestral repeat types and distributions. Amphibians are likewise underrepresented: only two anuran assemblies represent the entire class, making it impossible to draw robust inferences about repeat–gene density dynamics in Amphibia. Furthermore, unplaced scaffolds are excluded under our chromosome selection rules, so gene density on unassigned sequences does not contribute to our measures even when those sequences are large. Expanding high‐quality, chromosome‐level assemblies for underrepresented groups (especially those with extreme genome sizes or atypical karyotypes) will be critical for determining whether the correlations we observe in Mammalia, Actinopterygii, and Sauropsida hold across the full breadth of vertebrate diversity.

Finally, assembly quality and completeness remain key constraints to capturing true repeat landscapes and, consequently, accurate inferences of gene density distributions within genomes. Standard benchmarks, such as BUSCO completeness and N_50_, do not guarantee that repetitive regions are fully represented. Repetitive elements are challenging to fully assemble, and if they are underrepresented or fragmented during assembly, local gene density can be artificially inflated. Moreover, assessing gene annotation quality beyond BUSCO scores is difficult, meaning that even high‐quality assemblies can harbor undetected gaps in repeat‐dense regions. Alternatively, metrics such as the LTR Assembly Index (LAI; [36]) have been developed to assess the assembly quality of repetitive sequences. LAI depends on the accurate identification of intact LTR retrotransposons, and the authors demonstrated that structural prediction tools such as LTRharvest, which our pipeline employs, produce high false discovery rates [37]. In addition, LAI was originally conceived for use in plant genomes, and many vertebrate genomes, such as those of humans and zebrafish, fail to meet the LTR abundance threshold [36]. As long-read sequencing technologies and scaffolding algorithms continue to improve, revisiting these genomes with chromosome-level assemblies is likely to uncover additional, lineage-specific transposable elements or ancient repeats. More contiguous assemblies may refine repeat landscape analyses and uncover previously obscured genomic patterns.

In a broader context, our findings can be situated within the “accordion” model of genome size evolution [38], where the amount of DNA gained through TE expansion is counteracted by DNA loss through segmental deletion. Our results extend this framework by demonstrating that lineage-specific TE activity also affects the genome’s internal organization, particularly the proportional distribution of genes across chromosomes. This suggests that the accordion model, originally conceived to explain total DNA content, has a genomic architectural dimension warranting further investigation.

Future work may explore several open questions. First, we hypothesized that chromosomal rearrangements such as fissions, fusions, inversions, and translocations may reduce variation in gene density by mixing gene-rich and gene-poor regions; however, this mechanism remains to be explicitly tested. Phylogenetic analyses that pair rearrangement histories with shifts in homogeneity will be key to evaluating this prediction. Second, do the relationships between repeat expansion and gene density homogeneity hold across the full vertebrate tree, or do some lineages deviate in ways that challenge our expectations? Broadening this framework may reveal informative exceptions that refine our understanding of genome evolution.

## Supporting information

S1 Fig Standalone analysis with the predictors being the age and proportion of all genomic repeats, as well as the interaction between these terms. The lack of significant predictors with strong effects suggests the importance of the identity and behavior of specific repeats for gene density homogeneity. No aggregate predictors were identified as significant for Mammalia.
(TIF)

S1 File Species included in the study and filtering outcomes. Species were only used in model averaging when both gene density measures and repeat landscape data were available.
(CSV)

S2 File Standard and phylogenetic ANOVA results for gene density variation between Mammalia, Actinopterygii, and Sauropsida. (CSV)

S3 File Model averaging confidence set for all analyzed species, Mammalia only, Actinopterygii only, and Sauropsida only. (CSV)

S4 File Species excluded by Cook’s D in the analysis summarized by Fig 4. (CSV)
